# Long non-coding RNAs as prognostic markers in human breast cancer

**DOI:** 10.18632/oncotarget.7828

**Published:** 2016-03-01

**Authors:** Hairong Liu, Juan Li, Pratirodh Koirala, Xianfeng Ding, Binghai Chen, Yiheng Wang, Zheng Wang, Chuanxin Wang, Xu Zhang, Yin-Yuan Mo

**Affiliations:** ^1^ Cancer Institute, University of Mississippi Medical Center, Jackson, MS, USA; ^2^ Department of Oncology, Shandong Provincial Qianfoshan Hospital, Shandong University, Jinan, China; ^3^ Department of Clinical Laboratory, Qilu Hospital, Shandong University, Jinan, Shangdong Province, China; ^4^ Department of Biochemistry, University of Mississippi Medical Center, Jackson, MS, USA; ^5^ College of Life Science, Zhejiang Sci-Tech University, Hangzhou, Zhejiang, China; ^6^ Department of Urology, Affiliated Hospital of Jiangsu University, Jiangsu, Zhenjiang, China; ^7^ School of Computing, University of Southern Mississippi, Hattiesburg, MS, USA; ^8^ Center of Biostatistics and Bioinformatics, Department of Preventive Medicine, University of Mississippi Medical Center, Jackson, MS, USA; ^9^ Department of Pharmacology/Toxicology, University of Mississippi Medical Center, Jackson, MS, USA

**Keywords:** lncRNA, prognosis, breast cancer, biomarkers

## Abstract

Long non-coding RNAs (lncRNAs) have been recently shown to play an important role in gene regulation and normal cellular functions, and disease processes. However, despite the overwhelming number of lncRNAs identified to date, little is known about their role in cancer for vast majority of them. The present study aims to determine whether lncRNAs can serve as prognostic markers in human breast cancer. We interrogated the breast invasive carcinoma dataset of the Cancer Genome Atlas (TCGA) at the cBioPortal consisting of ~ 1,000 cases. Among 2,730 lncRNAs analyzed, 577 lncRNAs had alterations ranging from 1% to 32% frequency, which include mutations, alterations of copy number and RNA expression. We found that deregulation of 11 lncRNAs, primarily due to copy number alteration, is associated with poor overall survival. At RNA expression level, upregulation of 4 lncRNAs (LINC00657, LINC00346, LINC00654 and HCG11) was associated with poor overall survival. A third signature consists of 9 lncRNAs (LINC00705, LINC00310, LINC00704, LINC00574, FAM74A3, UMODL1-AS1, ARRDC1-AS1, HAR1A, and LINC00323) and their upregulation can predict recurrence. Finally, we selected LINC00657 to determine their role in breast cancer, and found that LINC00657 knockout significantly suppresses tumor cell growth and proliferation, suggesting that it plays an oncogenic role. Together, these results highlight the clinical significance of lncRNAs, and thus, these lncRNAs may serve as prognostic markers for breast cancer.

## INTRODUCTION

The human genome is actively transcribed. Of interest, protein-coding genes only account for ~2% whereas the rest of transcripts are non-coding RNAs including microRNAs and long non-coding RNAs (lncRNAs) [1]. MicroRNAs have been shown to play an important role in cancer initiation, progression and metastasis, and they may serve as potential biomarkers for cancer diagnosis and prognosis [2, 3]. Compared to well-studied microRNAs, lncRNAs are poorly characterized. To date, an overwhelming number of lncRNAs have been identified [4, 5]. Based on genomic organization and relationship to protein-coding genes, lncRNAs can be classified into five groups [6]: 1) sense; 2); antisense; 3) bidirectional; 4) intronic and 5) intergenic. Since lncRNA research is still at an early stage, the function for the vast majority of lncRNAs remains to be determined yet. In particular, little is known whether lncRNAs can serve as biomarkers for cancer diagnosis and prognosis.

Breast cancer is the second most common cancer in women in the U.S. based on the latest information from American Cancer Society (www.cancer.org). In 2015 about 231,840 new cases of invasive breast cancer will be diagnosed in women and about 40,290 women will die from breast cancer. Breast cancer is a heterogeneous disease with a large number of genetic alterations. For example, six subtypes have been identified based on gene expression profile and the phenotype. They are luminal A, luminal B, tumor enriched with human epidermal growth factor receptor 2 (HER-2), basal-like, normal-like and claudin-low subtype [7–10]. For example, luminal A is the most common subtype characterized by the expression of estrogen receptor (ER), progesterone receptor (PR), Bcl-2 and absence of HER-2. It accounts for 50-60% of the total breast cancer cases [8, 9]. The luminal B subtype is characterized by the expression of ER, PR and absence of HER-2. They can be differentiated from luminal A subtype on the basis of high Ki-67 staining which indicates higher proliferation rate [11]. The treatment for different subtypes of breast cancer is often different. However, the molecular pathogenesis of breast cancer remains poorly defined due to its heterogeneity. Although expression of group of specific microRNAs can be associated with cancer subtype [12], it is not clear about the role of lncRNAs in this aspect, and their clinical implication remains to be determined yet.

In the present study, we interrogated the breast invasive carcinoma dataset of the Cancer Genome Atlas (TCGA) at cBioPortal, and identified three lncRNA signatures that can predict overall survival (OS) or recurrence. Furthermore, we characterized one lncRNA from the signatures, LINC00657, by knockout and cell culture models and demonstrated that LINC00657 plays an oncogenic role in breast cancer.

## RESULTS

### Genetic alterations of lncRNAs and patient overall survival (OS)

**Figure 1:**
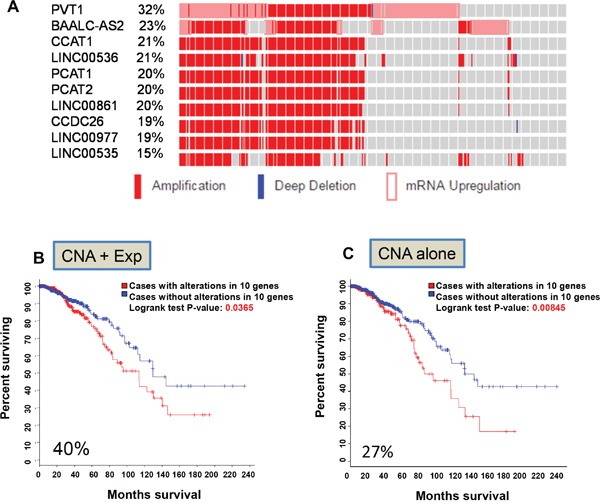
Identification of the first lncRNA signature associated with overall survival **A.** Top 10 lncRNAs based on alteration frequency primarily due to CNA and upregulation. **B.** Kaplan–Meier curve for OS due based on alterations of these 10 lncRNAs (CNA and upregulation). **C.** Kaplan–Meier curve based on alterations of these 10 lncRNAs (CNA alone).

Long non-coding RNAs (lncRNAs) are a very large mixed group of non-coding RNAs that are arbitrarily defined as larger than 200 bp in length [13]. Hence, we performed a primary search starting with 2730 lncRNAs primarily consisting of long intervening non-coding RNAs (lincRNAs) and anti-sense lncRNAs (Table S1). The cBioPortal recognized 2,553 of them as valid names (Table S2). Although the vast majority of them had no alterations, there were still quite a few lncRNAs with various genetic alterations. For example, 577 lncRNAs had alterations with alteration frequency ranging from 1% to 32% (Table S2). Forty five lncRNAs had alterations in 10% cases or above (Table S2); 10 of them (PVT1, CCAT1, LINC00536, PCAT1, PCAT2, LINC00861, CCDC26, LINC00977, BAALC-AS2 and LINC00535) accounted for a total of 40% cases (Figure 1A). These lncRNAs were all on chromosome 8q (Figure S1A) and heavily overlapped, primarily due to copy number alteration (CNA). Among them, PVT1 had the highest frequency (32%) and the lowest was LINC00535 with 16% frequency. Of note, not all amplifications led to upregulation; instead only 2 of them, PVT1 and BAALC-AS2, had upregulation (Figure S1B). No downregulation or mutations (missense or truncating mutation), were found. Importantly, each of these lncRNAs except for PVT1 revealed significant associations with OS (Table S2). When all 10 lncRNAs were combined, their alterations were also significantly associated with OS (Logrank test p = 0.0365) (Figure 1B).

Since this primary search included all three parameters (mutation, CNA and RNA expression) and most of alterations were due to amplification and upregulation (Figure S1B), we determined whether a single feature is able to predict patient outcomes. This search identified 27% frequency for CNA alone and they were associated with OS and logrank test p value was 0.00845 (Figure 1C) among 960 samples. However, when expression was used as a sole criterion, none of these 10 lncRNAs was able to predict outcomes with a significant p value (not shown). Of interest, all of them were located on chromosome 8q (from 8q22 to 8q24) (Table S3).

In this regard, chromosome 8q24 has been identified in a large scale study as the most frequently amplified region linked to different cancers [14, 15]. The well-known oncogene Myc is at chromosome 8q24.21. To determine whether these lncRNAs are closely associated with Myc, we chose 2~3 genes from each chromosome band from 8q21.13 to 8q24.3 to search the alternations of putative copy number. The farer from Myc, the smaller the changes of copy number become (Figure S2A & B), suggesting that 8q24.21 is the center of CNA. Although 8q24 is believed to be a susceptible region, our analysis suggested that CNA for these lncRNAs may involve an entire region from 8q11.1 to 8q24.3, and it is associated with OS.

### Alterations of lncRNAs in RNA expression and OS

**Figure 2:**
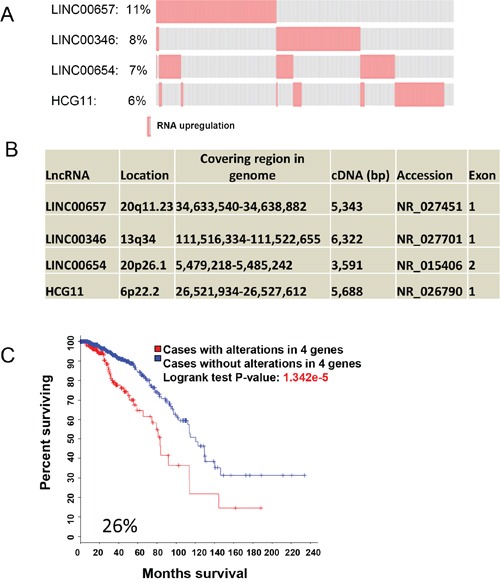
LncRNA signature for OS based on RNA expression **A**. Upregulation of LINC00657, LINC00346, LINC00654 and HCG11 with alteration frequency. **B.** Organizations of these 4 lncRNAs and chromosome locations. **C.** Upregulation of these 4 lncRNAs is significantly associated with OS as shown by Kaplan–Meier curve.

CNA accounts for the large part of genetic alterations, and these data are obtained largely through genomic DNA copy number arrays [16]. Given that deep sequencing becomes widely used and it provides more valuable information, we tried to determine whether deregulation of lncRNA expression (RNA-seq) alone can predict patient survival outcomes. A secondary search for significant predictors was performed in 1098 samples. Compared to CNA, alterations of gene expression were less frequent. For example, only 8 lncRNAs were above 10% alteration frequency and 95 lncRNAs revealed alternations in above 5% alternation frequency (Table S2). Using Onco Query Language (OQL) EXP>2, we found that 275 of 1098 cases had upregulation for LINC00657, LINC00346, LINC00654 and HCG11; total alteration frequency for these 4 lncRNAs was 26% (Figure 2A). Unlike the first lncRNA signature primarily due to CNA that all lncRNAs were clustered (Figure 1A), these 4 lncRNAs were well separated (Figure 2B). For example, they were either on different chromosomes or they were at least 29 Mb apart if they are on the same chromosome. Together, the association between lncRNA upregulation and OS was highly significant (p = 1.432e-5) (Figure 2C). In contrast to the first signature, CNA of these 4 lncRNAs had no association with OS (Figure S3). Individually, LINC00657 revealed 11% frequency of alterations in 1098 samples and was the highest among these four lncRNAs. Its upregulation was significantly associated with OS (Figure S4A). The upregulation frequency and p values for the other 3 lncRNAs association with OS were shown in Figure S4B~D. Of note, upregulation of these lncRNAs had no association with recurrence (not shown).

### A lncRNA signature for recurrence

Recurrence is a major concern for cancer survivors. Since the first two signatures were not able to predict recurrence, next we searched for a lncRNA signature for recurrence. There were 68 cases of recurrence in this cohort (Table S4). We found that upregulation of 18 lncRNAs with p value <0.05 was associated with recurrence (Table S5). To determine the optimal number of lncRNAs for prediction of recurrence, we adopted a stepwise forward selection approach based on Cox model to identify lncRNAs significantly associated with recurrence. 1) All variables were individually included in the Cox model on recurrence. The variable with smallest p value and below the 5% threshold was first selected. 2) First, the remaining variables were evaluated in the Cox model on recurrence with previously selected variable(s). Next, the variable with smallest p value and below 5% threshold entered the model. Finally, if any variable became insignificant after inclusion of the new variable, the insignificant variable was removed. We repeated step 2 until none of the remaining variables having p value less than 0.05. 3) All possible models based on the selected variables are evaluated by AIC. The model with the smallest AIC value was determined as the optimal model.

**Figure 3:**
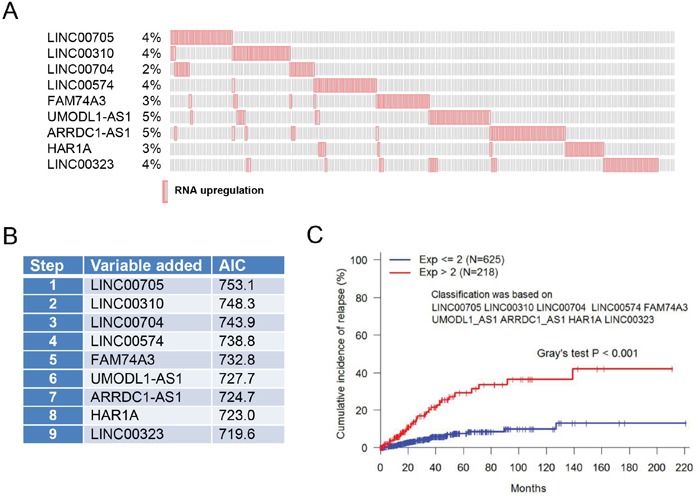
LncRNA signature for recurrence based on RNA expression **A.** Upregulation of 9 lncRNAs with alteration frequency. **B.** Nine lncRNAs are selected as a signature for prediction of recurrence based on AIC estimation. **C.** Upregulation of these 9 lncRNAs is significantly associated with recurrence.

Based on AIC estimation, 9 of 18 lncRNAs were selected as a signature for prediction of recurrence (Figure 3A & 3B). Individually, they were upregulated with alteration frequency of 2~5%, and together about 28% in a total of 1105 samples (Figure 3A). Thus, this Cox based search generated a third lncRNA signature consisting of LINC00705, LINC00310, LINC00704, LINC00574, FAM74A3, UMODL1-AS1, ARRDC1-AS1, HAR1A and LINC00323 (Figure 3B). Upregulation of this signature (exp > 2.0) was found in 228 of total 943 follow up cases; it was distinctly different from the no upregulation group (exp ≤ 2.0) with Gray's test p < 0.001 (Figure 3C).

### Association of lncRNA signatures with clinicopathologic features

**Figure 4:**
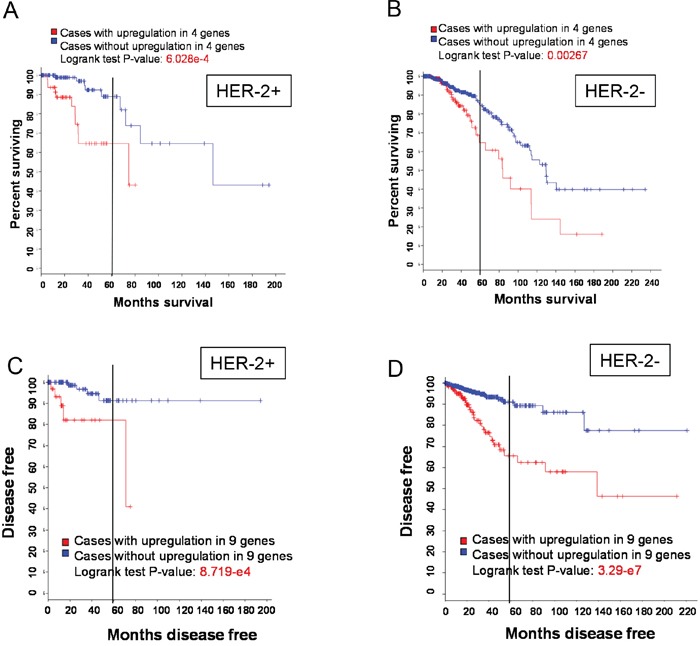
HER-2 status and OS or recurrence **A & B.** A poorer OS with upregulation of signature 2 in HER-2 positive patients than in HER-2 negative patients. **C & D.** A higher recurrence with upregulation of signature 3 in HER-2 negative patients than in HER-2 positive patients.

Based on RNA expression we identified 2 signatures (signature 2 and 3) consisting of 13 lncRNAs capable of predicting OS or recurrence. Thus, we determined any association of these lncRNAs with clinicopathologic features including age, tumor stage, metastatic status, ethnicity as well as the stature of ER, PR and HER-2. Among them, we found no significant association with age, tumor stage, metastatic status or ethnicity. However, their expression was associated with HER-2. For example, although expression of signature 2 (LINC00657, LINC00346, LINC00654 and HCG11) was associated with overall survival in both HER-2 positive and HER-2 negative cases, the logrank test P-value was much smaller for HER-positive than for HER-2 negative cases (Figure 4A & 4B). Furthermore, in HER-2 positive patients 5 year survival rate was about 90% for cases with upregulation of this signature compared to ~65%. In contrast, in HER-2 negative patients 5 year survival rate was ~85% for cases with upregulation of this signature compared to ~65% for cases without upregulation, implying poorer prognosis for HER-2 positive patients with upregulation of this signature than for HER-2 negative patients. For signature 3 we found that 5 year survival rate was about 90% for cases with upregulation of this signature compared to ~80% for cases without upregulation (Figure 4C). In contrast, in HER-2 negative patients 5 year survival rate was ~90% for cases with upregulation of this signature compared to ~65% for cases without upregulation (Figure 4D), suggesting poorer prognosis for HER-2 negative patients with upregulation of this signature.

Next, we examined associations of alterations for individual lncRNAs derived from signature 2 and 3 with ER, HER-2 or triple negative. Based on initial p values, 11 of 13 lncRNAs were associated with ER status (Table S6). For example, in ER positive patients, LINC00657 regulation accounted for 60%, but no upregulation accounted for 71% In HER-2 positive patients, 34% had upregulation of LINC00654 upregulation accounted for 34%, no upregulation accounted for 66%. In the triple negative group, alteration for each of 6 lncRNAs (HCG11, LINC00310, LINC00704, UMODL1-AS1, ARRDC1-AS1 and HAR1A) was significantly higher than no alteration (Table S6).

Since the alteration frequency for each of these lncRNA varies, along with 3 clinical conditions, to integrate these variations, we applied the Bonferroni correction to control the overall type I error rate at 5%. The Bonferroni-adjusted P-value was calculated by multiplying the raw P-value by 39. Table 1 shows the adjusted P-values for associations between alterations of lncRNAs and ER, HER-2 and triple negative status. After adjustment, none of the lncRNAs was significantly associated with HER-2 status. HCG11 and UMODL1-AS1 were both significantly associated with ER and triple negative status. Status of ER was also significantly associated with a few other lncRNAs, including LINC00346, LINC00654, LINC00704, and ARRDC1-AS1.

**Table 1:** Alterations of lncRNAs associated with ER, PR and HER-2

		ER		HER-2		Triple negative	
		Positive N (%)	Bonferroni-adjusted *P* value	Positive N (%)	Bonferroni-adjusted *P* value	Positive N (%)	Bonferroni-adjusted *P* value
LINC00657	Alteration (N=114)	68 (60%)	0.479	21 (18%)	1.000	20 (18%)	1.000
	No alteration (N=845)	603 (71%)		164 (19%)		163 (27%)	
LINC00346	Alteration (N=72)	14 (19%)	**<0.0001**	10 (14%)	1.000	16 (22%)	1.000
	No alteration (N=887)	657 (74%)		175 (20%)		167 (19%)	
LINC00654	Alteration (N=67)	23 (34%)	**<0.0001**	23 (34%)	0.129	13 (19%)	1.000
	No alteration (N=892)	648 (73%)		165 (18%)		170 (19%)	
HCG11	Alteration (N=55)	14 (25%)	**<0.0001**	7 (13%)	1.000	33 (60%)	**<0.0001**
	No alteration (N=904)	657 (73%)		178 (20%)		150 (16%)	
LINC00705	Alteration (N=29)	14 (48%)	0.514	4 (14%)	1.000	9 (31%)	1.000
	No alteration (N=930)	657 (70%)		181 (19%)		174 (19%)	
LINC00310	Alteration (N=36)	18 (50%)	0.565	5 (14%)	1.000	15 (41%)	0.063
	No alteration (N=923)	653 (71%)		180 (20%)		168 (19%)	
LINC00704	alteration (N=19)	2 (11%)	**<0.0001**	7 (37%)	1.000	8 (42%)	0.653
	No alteration (N=940)	669 (71%)		178 (19%)		175 (19%)	
LINC00574	Alteration (N=36)	31 (86%)	1.000	4 (11%)	1.000	3 (8%)	1.000
	No alteration (N=923)	640 (69%)		181 (20%)		180 (20%)	
FAM74A3	Alteration (N=36)	25 (69%)	1.000	4 (11%)	1.000	6 (17%)	1.000
	No alteration (N=923)	646 (70%)		181 (20%)		177 (19%)	
UMODL1-AS1	Alteration (N=42)	17 (40%)	**0.002**	3 (11%)	1.000	18 (43%)	**0.014**
	No alteration (N=917)	654 (71%)		182 (20%)		165 (18%)	
ARRDC1-AS1	Alteration (N=31)	12 (39%)	**<0.0001**	4 (13%)	1.000	13 (42%)	1.000
	No alteration (N=928)	659 (71%)		184 (20%)		170 (18%)	
LINC00323	Alteration (N=37)	24 (65%)	1.000	4 (11%)	1.000	10 (27%)	1.000
	No alteration (N=922)	647 (70%)		181 (20%)		173 (19%)	
HAR1A	Alteration (N=26)	24 (92%)	0.346	3 (12%)	1.000	1 (3%)	1.000
	No alteration (N=933)	647 (69%)		182 (19%)		182 (19%)	

### LINC00657 is a potential oncogenic gene

**Figure 5:**
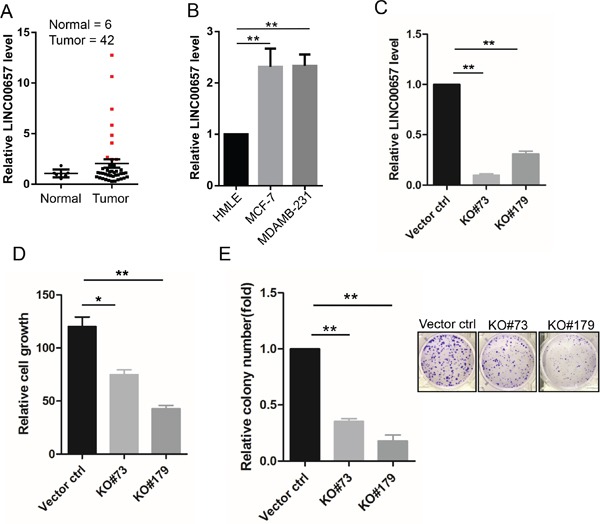
Upregulation of LINC00657 in breast cancer and its promotion of cell growth and proliferation **A.** Expression of LINC00657 in the OriGene breast cancer tissue cDNA array, as determined by qPCR. **B.** LINC00657 is upregulated in breast cancer cells (MCF-7 and MDA-MB-231) as compared to non-malignant breast cells (HMLE). **C.** Expression of LINC00657 in KO cells as compared to vector control. **D & E.** LINC00657 KO suppresses cell growth, as detected by MTT assays and colony formation, as detected by clonogenic assays. Values in B, C, D, E and F are SEM (n = 3). *, p < 0.05; **, p < 0.01.

Upregulation of these lncRNAs is associated with overall survival or recurrence, suggesting that they may play an oncogenic role. Hence, we selected LINC00657 to determine its effect on breast cancer because alteration of LINC00657 with RNA expression occurred at 11% frequency, the highest among the 4 lncRNA signature (Figure 2A). Moreover, LINC00657 has been recently shown to play a role in genomic stability [17]. To this end, we first profiled breast cancer cDNA arrays from OriGene consisting of 43 tumor and 5 normal. In 9 of 43 samples (21%) LINC00657 expression level was above a 2-fold of the mean expression level (Figure 5A). Consistent with this finding, we found that LINC00657 was also upregulated in breast cancer cell lines MCF-7 and MDA-MB-231 cells as compared to non-malignant HMLE cells (Figure 5B).

Therefore, we knocked out LINC00657 by CRISPR/Cas9 system (Figure S5) in LM-4142, a derivative cell line from MDA-MB-231 [18]. We selected two of them for further characterization (Figure 5C, RT-PCR data). MTT assays indicated that LINC00657 KO caused significant reduction of cell growth (Figure 5D). Consistent with this result, clonogenic assays indicated that the number of colonies was much smaller in KO cells than in vector control (Figure 5E). These results suggest that LINC0657 impacts tumor cell proliferation and cell growth and thus, LINC00657 is a potential oncogene gene.

## DISCUSSION

Despite large numbers of human lncRNAs identified so far, little is known whether they can serve as markers for cancer diagnosis/prognosis. The present study focus on a special group of lncRNAs from HGNC to interrogate the breast invasive carcinoma dataset (provisional) at the cBioPortal. This dataset consists of 960 complete tumor samples and over 1000 samples with RNA expression (RNA-seq) data. From over 2700 lncRNAs we identify three specific lncRNA signatures: 1) lncRNA signature based on alterations of CNA and RNA expression that is associated with OS; 2) lncRNA signature based on alterations of RNA expression alone that is associated with OS; and 3) lncRNAs based on alternations of RNA expression alone that is associated with recurrence. Together, our study suggests that lncRNAs are potential prognostic markers for breast cancer and thus further investigations of these lncRNAs are warranted.

Early work of biomarker discovery in breast cancer focuses on protein-coding genes such as Ki-67, ER, PR, and HER-2 [19]. For example, expression of ERα is a well-established prognostic factor in breast cancer patients [20]. Other molecular biomarkers include p53, p14^ARF^, cyclin D1, cyclin E, TBX2/3, and VEGF [21] and genetic mutations such as BRCA1 and BRCA2 [22]. Furthermore, several multigene signature have been developed in recent years to predict risk of breast cancer recurrence after a primary diagnosis [23, 24]. In particular, Oncotype-Dx, (Genomic Health) consisting of 21 genes, can provide treatment recommendations in conjunction with risk of recurrence [25]. Compared to protein-coding genes, the number of lncRNAs is much larger [26], and thus, lncRNAs should be a rich source for biomarker discovery. Therefore, potential lncRNA signatures would at least complement the existing biomarkers, providing additional information that may help improve the predictability.

Interrogation of this dataset provides a new perspective on the role of lncRNAs as biomarkers for breast cancer diagnosis prognosis. Although early studies have shown that lncRNAs may serve as prognostic markers, their functional role in prognosis may vary even with the same lncRNA. HOTAIR is one of the early identified lncRNAs and plays a significant role in gene regulation through remodeling chromatin structures [27]. Importantly, elevated expression of HOTAIR was reported in both primary and metastatic breast cancer and it is a significant predictor of subsequent metastasis and death [28]. Moreover, Sorensen et al showed that HOTAIR is a prognostic marker of metastasis in ER positive breast cancer from 164 patients by microarray analysis [29]. However, Gokmen-Polar et al [30] reported that HOTAIR was only a poor prognostic indicator in ER negative breast cancer from 952 patients in TCGA database. In contrast, high HOTAIR expression had lower risks of relapse and mortality than those with low HOTAIR expression through 336 breast cancer patients [31]. Several possibilities may contribute to the conflicting results. First, detection methods are different in these studies, including qRT-PCR, microarray analysis and HOX tiling array. Second, the cut-off value of high and low HOTAIR expression as well as ER status in these studies may be different. Third, there is a significant heterogeneity in these studies, such as the races, the tumor sizes and the clinical stages. These factors may also explain why HOTAIR does not meet the cut as a prognostic marker in our study. Alternatively, the signatures identified in this study may be more potentially as diagnostic markers than HOTAIR.

Although the role of these lncRNAs within three signatures in breast cancer remains to be determined yet, their association with cancer death or recurrence may suggest that they play an oncogenic role. Since the first signature consists of lncRNAs primarily due to CNA, they are clustered in close association with Myc. Thus, the possible oncogenic role, if any, is likely from Myc. In particular, not all amplification cases cause upregulation of their corresponding lncRNAs. On the other hand, both the second and third signatures consist of lncRNAs solely due to upregulation and they are well separated, and the upregulation of these lncRNAs may suggest that they may function as drivers. In support of this notion, we showed that LINC00657 may play an oncogenic role. For example, LINC00657 KO suppresses cell growth and proliferation. Thus, LINC00657 may serve as not only a biomarker, but also a potential therapeutic target.

Given the clinical potential of these lncRNAs identified in this this study, it would be interesting to determine whether they can also be detected in the circulation system. In support of this possibility, recent studies suggest that serum lncRNAs may serve as potential biomarkers for hepatocellular carcinoma and breast cancer [32–34]. In addition, lncRNAs can also be present in urine. For example, prostate cancer antigen 3 (PCA3 or DD3) is a lncRNA that is highly overexpressed in prostate cancer. In particular, presence of PCA3 in urine can predict prostate biopsy outcome [35]. Therefore, further characterization of them is warranted.

Finally, in addition to biomarker discovery, this study provides an example of how researchers with little knowledge in bioinformatics can utilize the existing public data for lncRNA research. Given the overwhelming number of lncRNAs, a challenge for average research laboratories is how to focus on lncRNAs with clinical relevance. The cBioPortal might be a good start point before launching real experiments. The portal currently contains data from 105 cancer genomics studies and a variety of cancer types. Since the dataset we used in this study is still provisional, the number of samples keep increasing. We expect that this may further enhance the predictability in future.

## MATERIALS AND METHODS

### Database search

The cBioPortal (http://www.cbioportal.org) is an open-access resource for interactive exploration of multidimensional cancer genomics data sets, currently providing access to data from more than 5,000 tumor samples from 105 cancer studies in the TCGA pipeline [36, 37]. Although there are overwhelming numbers of human lncRNAs reported from databases, the nomenclature of lncRNAs is still incomplete. In this study we focused on those lncRNAs by HUGO gene nomenclature committee (HGNC) (http://www.genenames.org/) where we downloaded a total of 2730 lncRNAs (http://www.genenames.org/cgi-bin/statistics) (Table S1) for our analysis when this study was initiated.

Genomic data types integrated by cBioPortal included somatic mutations, DNA copy number alterations (CNAs), mRNA and microRNA expression, DNA methylation, protein abundance, and phosphoprotein abundance. The portal contained several sets of samples for breast cancer. From Breast Invasive Carcinoma dataset (TCGA, provisional) as shown in Figure S6, we chose 1) “All complete tumors with 960 samples (when the primary search was performed) or 2) tumors with mRNA data (RNA-seq V2) from 1098 or 1105 samples. The primary search parameters included mutations, CNA from GISTIC and mRNA expression (RNA seq data) with the default setting. For the secondary search, we focused on RNA seq data.

### Statistical analysis

All available lncRNAs were sorted by alteration frequency at the cBioPortal. Those lncRNAs with significant log-rank p values were entered the candidate pool to be considered for further selection. The forward selection was performed among the pool of candidates by using the Cox model on progression. The Akaike information criterion (AIC) was further evaluated for the models that were finalized at each step of forward selection. The model with smallest AIC value was chosen as the final model and the lncRNAs in the final model were identified as predictors of progression. The study cohort of breast cancer patients were divided into those with high expression on any of the predictors and those with normal expressions on these predictors. Progression and death without progression were treated as competing risks. The cumulative incidence of progression was estimated in the aforementioned two patient subgroups. The difference in cumulative incidence of progression between two subgroups was evaluated by the Gray's test [38]. P values less than 5% were determined as significant. The statistical analyses were performed using the SAS software (version 9.3, the SAS institute) and R package “cmprsk” for competing risks analysis.

### Cell culture

LM-4142 cells originally derived from MDA-MB-231 were kindly provided by Dr. Joan Massagué (Memorial Sloan-Kettering Cancer Center) as described previously [18]. Cells were cultured in RPMI-1640 medium with 10% FBS and 2mM glutamine. All culture media were supplemented with 100 units/mL penicillin and 100 μg/mL streptomycin.

### qRT-PCR

Total RNA was isolated using Direct-zol™ RNA MiniPrep Kit (Zymo Research, Irvine, CA) as suggested by manufacturer. Reverse transcription was carried out by using RevertAid Reverse Transcriptase (Fisher Scientific) and random primer mix (New England BioLabs, Ipswich, MA). The expression of lncRNAs was detected by quantitative RT-PCR (qRT-PCR) using SYBR Green method. Analysis of qRT-PCR was performed as described previously [39].

### Construction of plasmids

The high fidelity Phusion enzyme from Fisher Scientific (Pittsburgh, PA, USA) was used to amplify DNA fragments by PCR for cloning purpose. Dual gRNA targeting the entire exon of LINC00657 and the corresponding donor were constructed using the same method as described previously [40]. Dual gRNA was designed using WU-CRISPR [41] and their sequences were listed in Table S1. To increase the frequency of selection of complete knockout clones, we constructed a donor vector carrying left and right arm homologous to the flanking regions of the targeting sites. PCR was performed using human genomic DNA as a template and primer sets LINC00657-left-Spe I-5.1 and LINC00657-left-Spe I-3.1 (left arm), and LINC00657-right-Sal I-5.1 and LINC00657-right-Sal I-5.1 (right arm). These two fragments were sequentially cloned into donor vector at Spe I and Sal I sites as described previously [40]. All amplified fragments were verified by DNA sequencing.

### Knockout of LINC00657

LINC00657 has a single exon with 5,378 bps in length. We used a dual gRNA approach [40] to knock out LINC00657 by CRISPR/Cas9 system [42]. A donor vector carrying ~700 bp left or right arm derived from the outside regions of LINC00657 was used to facilitate selection of knockout (KO) clones. The dual gRNA construct carrying Cas9 and donor vector were introduced into LM-4142 cells, respectively, by transient transfection. As a control, everything was same except that the dual gRNA is an empty vector. One week later, the transfected cells were subject to puromycin selection; and surviving cells were sorted by FACS based on GFP signal into 96-well plates and then expanded. Initial identification of knockout clones was carried out by genomic PCR, followed by qRT-PCR, as described previously [40].

### MTT assays

MTT assay was performed to determine the effect of LINC00657 on cell growth in 96-welll plates as described previously [43].

### Clonogenic assays

To determine the clonogenic survival of LINC00657 KO cells, cells from either vector control or LINC00657 KO were seeded on 6-well plates at 1,000 cells/well. At 10 days after seeding, colonies were fixed and stained with 0.1% crystal violet.

## SUPPLEMENTARY FIGURES AND TABLES















## Abbreviations

lncRNA: long non-coding RNA
lincRNA: long intergenic non-coding RNA
TCGA: The Cancer Genome Atlas
OS: overall survival
CNA: copy number alteration
