# Supplementary material for: Long non-coding RNAs as prognostic markers in human breast cancer
Source: Oncotarget. 2016 Mar 1;7(15):20584–96. doi: 10.18632/oncotarget.7828 (PMC4991477; doi:10.18632/oncotarget.7828)
Supplement: Supplementary file 1 [file oncotarget-07-20584-s001.pdf]

SUPPLEMENTARY FIGURES AND TABLES

A

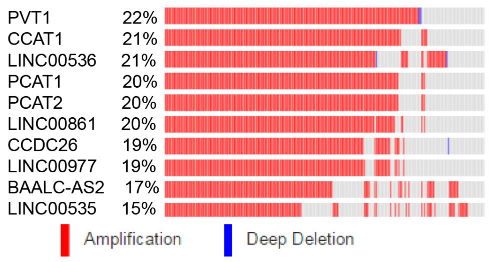

B

LncRNA	Location	Region on chromosome	cDNA (bp)	Accession	Exon
LINC00535	8q22.1	94,358,695---94,712,661	1,924	NR_033858	11
BAALC-AS2	8q22.3	104,145,191---104,153,570	496	NR_027071	2
LINC00536	8q22.3	116,962,736---117,337,297	2,692	NR_046215	14
LINC00861	8q24.3	126,953,376---126,963,441	5,338	NR_038446	2
PCAT1	8q24.21	128,025,399---128,033,259	1,992	NR_045262	2
PCAT2	8q24.21	128,084,939---128,094,466	575	NR_119373	4
CCAT1	8q24.21	128,219,629----128,231,333	2,795	NR_108049	2
PVT1	8q24.21	128,806,803----128,952,702	1,716	NR_003367	8
LINC00977	8q24.21	130,228,713---130,253,486	1,164	NR_033916	3
CCDC26	8q24.21	130,363,940---130.365.228	1,666	NR_130920	4
**MYC**	**8q24.21**	**128.747.765---128.751.018**			

C

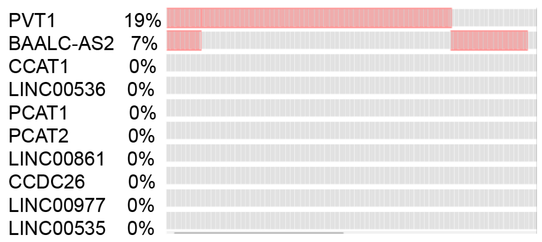

D

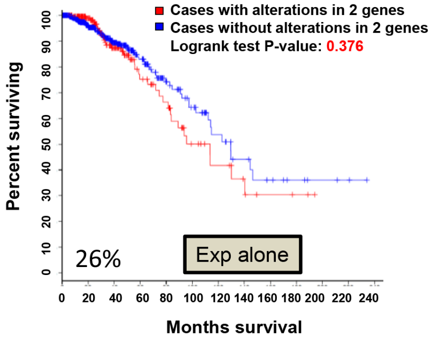

Supplementary Figure S1: Alterations in copy number and RNA expression for lncRNAs associated with poor OS. A. Relative alteration frequency of copy number for 10 lncRNAs. **B.** Listed 10 lncRNAs with location, cDNA size and accession numbers. **C.** Only 2 lncRNAs (PVT1 and BAALC-AS2) revealed alterations of RNA expression. **D.** Association between expression of PVT1 and BAALC-AS2 and OS is not significant.

A

B

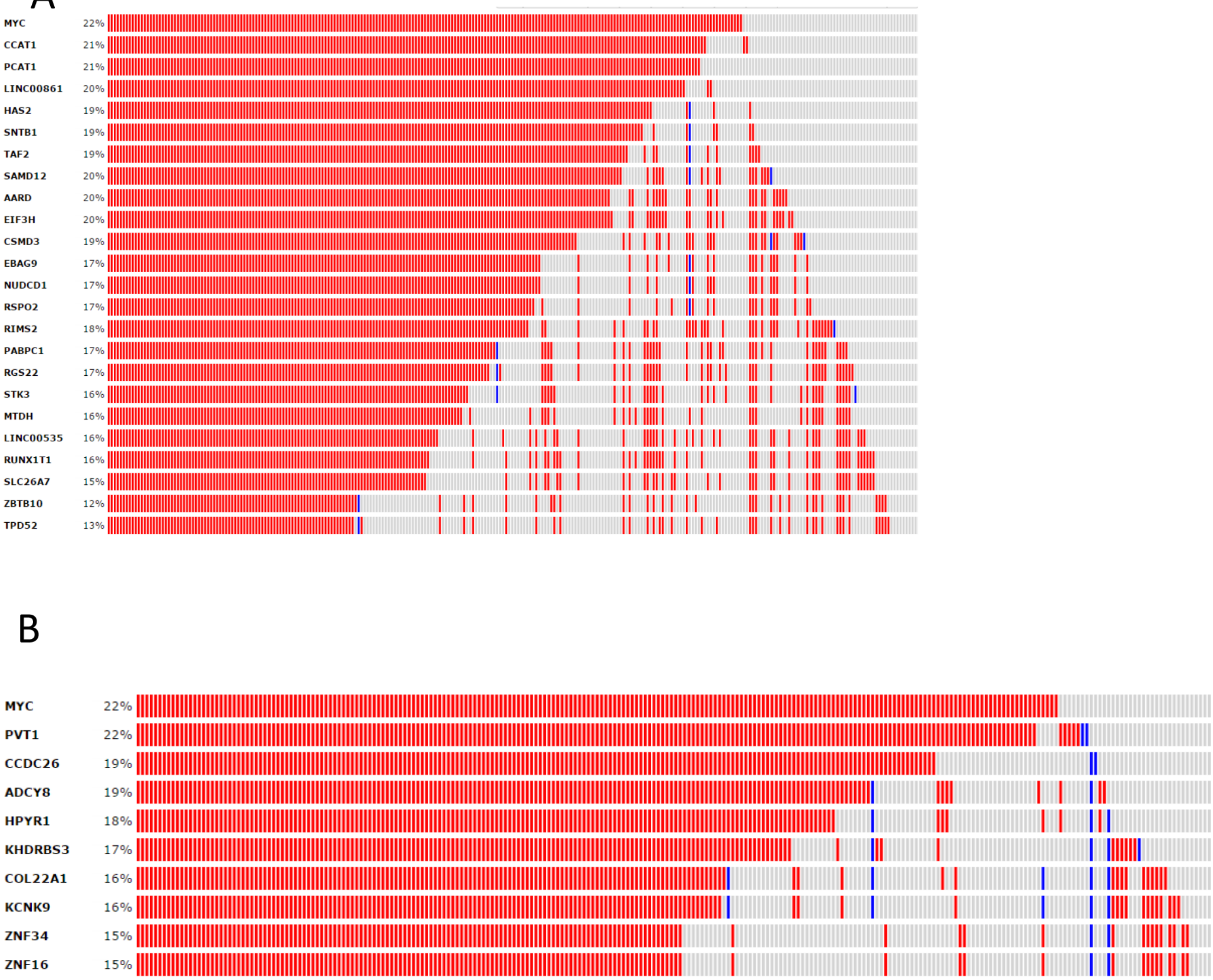

Supplementary Figure S2: Alterations in copy-number of lincRNAs related to Myc. A. Relative alteration frequency of copy number toward right side of Myc. **B.** Relative alteration frequency of copy number toward left side of Myc.

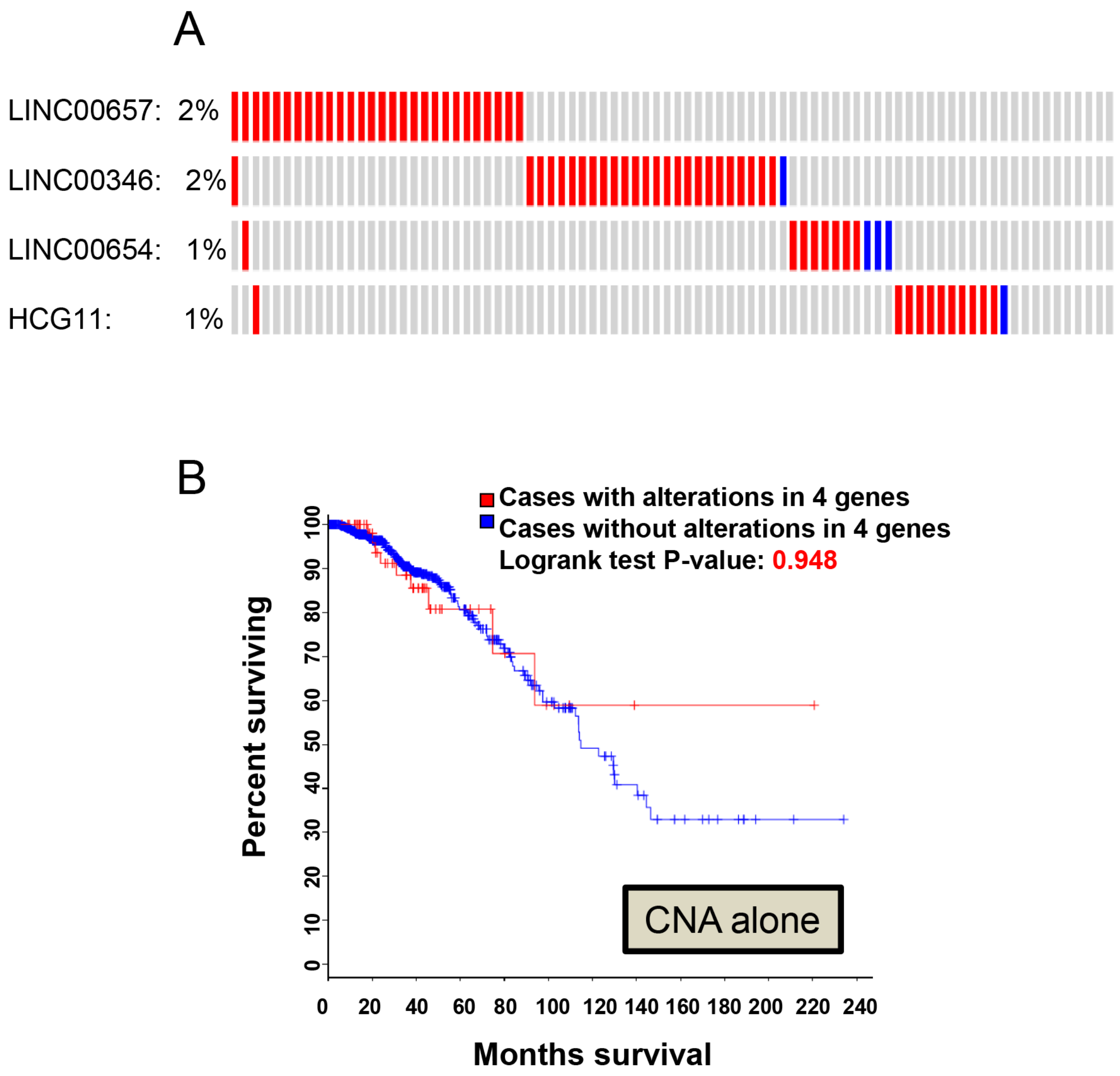

Supplementary Figure S3: Alterations in copy number of lncRNA signature 2. A. Relative frequency of CNA for 4 lncRNAs. **B.** Association between CNA and OS is not significant.

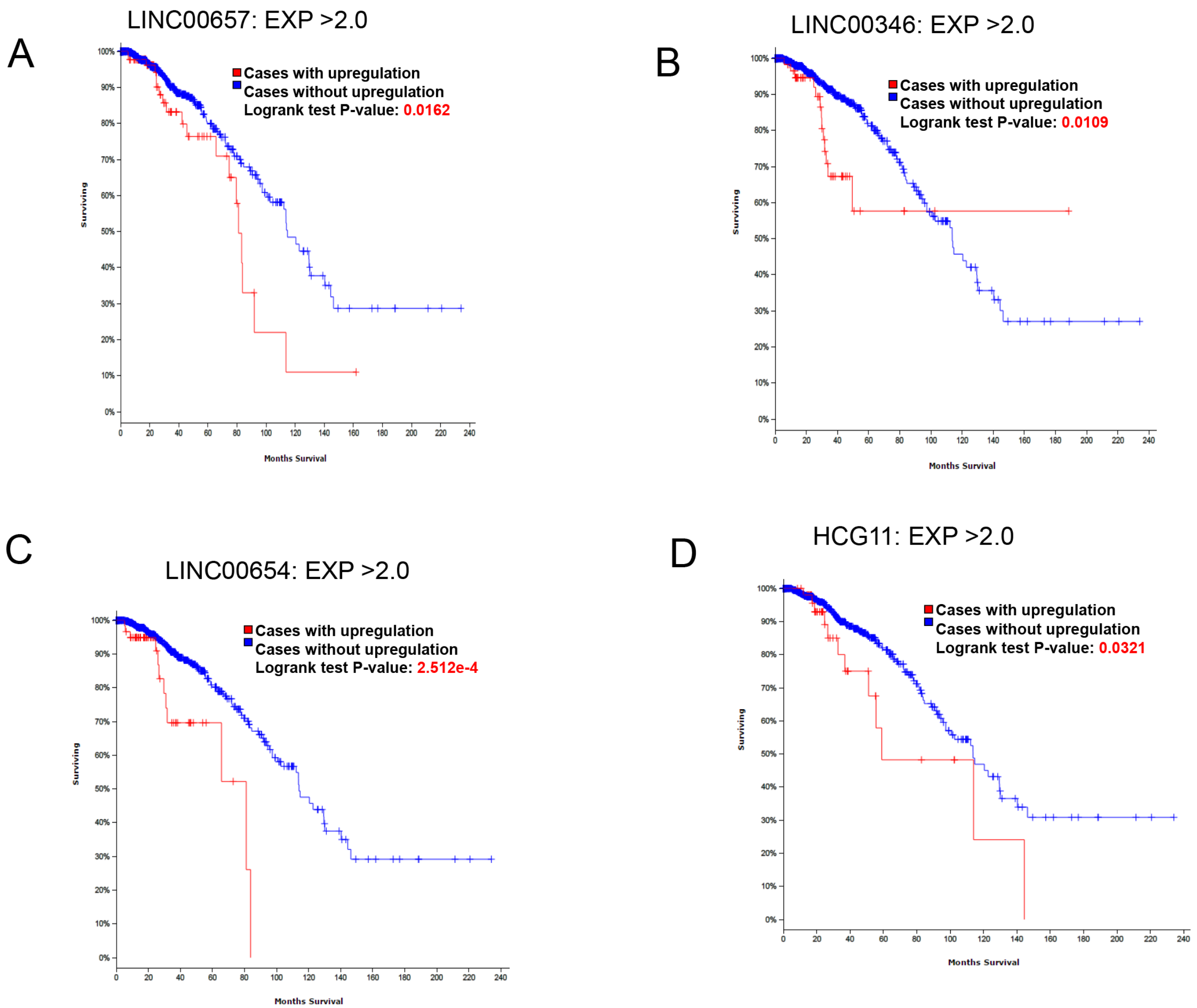

Supplementary Figure S4: Association of 4 individual lncRNAs with OS.

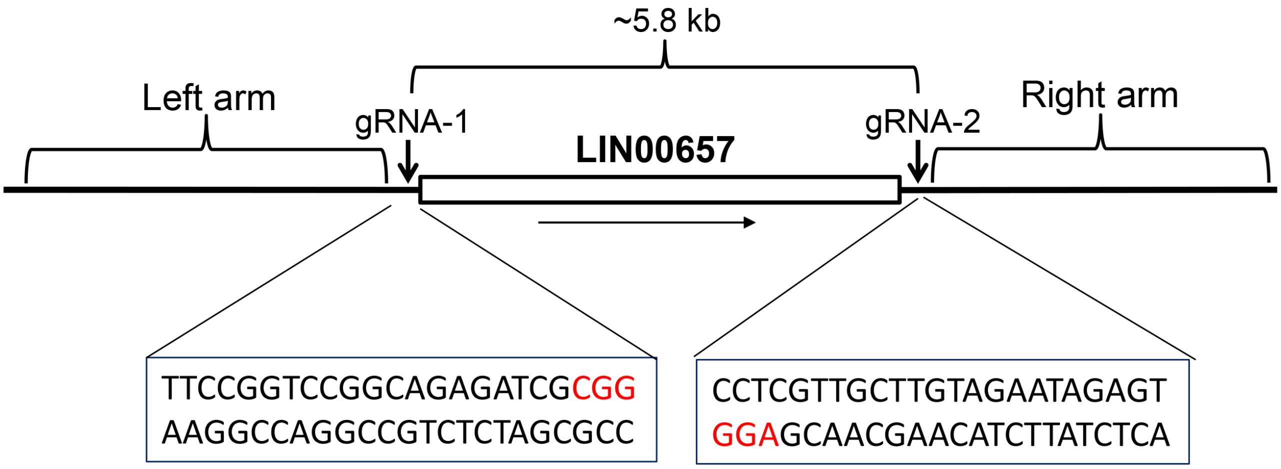

Supplementary Figure S5: A strategy for generation of LIN00657 knockout by CRISPR/Cas9. Two gRNA sequences are shown in bottom; PAM motifs are shown in red.

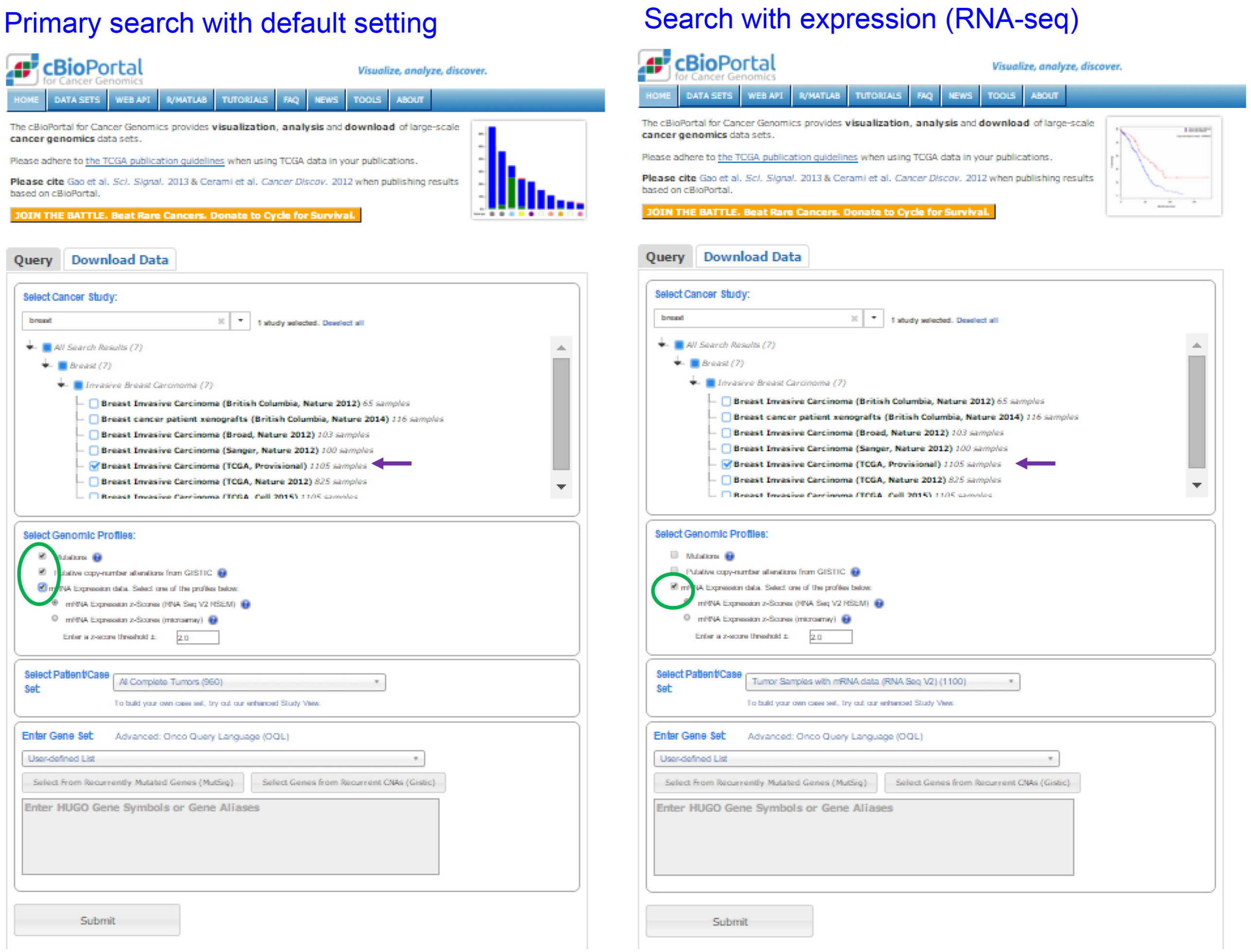

Supplementary Figure S6: Datamining with primary search with default setting and search for expression only (RNA-seq).

Supplementary Table S1: lncRNAs used for primary search

See Supplementary File 1

Supplementary Table S1a: Primers used in this study

For detection	
LINC00657-RT-5.1	AAAGAGGTTGCCGACGTATG
LINC00657-RT-3.1	CAGGTCTTCCAGCTCCATGT
LINC00657-RT-5.2	GCAGGAGAATCGCTTGAACT
LINC00657-RT-3.2	ATTCCCAAATGCAACCACTC
For dual gRNA	
LINC00657-T1A	TTCCGGTCCGGCAGAGATCG
LINC00657-T2A	ACTCTATTCTACAAGCAACG
For donor vector	
LINC00657-right-Sal1-5.1	TCTTATCATGTCTGGTCGACTCCTTCATGCTTGGGCTCTG
LINC00657-right-Sal1-3.1	TTGATATCACCGGTGTCGACGCTGTGTGTCAGGCATTACG
LINC00657-left-Spe-5.1	CGACGGCCAGTGAAACTAGTCCCTGGCATAAGTCCCTATC
LINC00657-left-Spe-3.1	CCTGTACAGGTACCACTAGTGACTTGGCAGTTCTCTGCGC

Supplementary Table S2: Alterations of lncRNAs and their frequeny

See Supplementary File 2

Supplementary Table S3: Distribution of lncRNAs aroung 8q24

See Supplementary File 3

Supplementary Table S4: Cases with recurrence in red

See Supplementary File 4

Supplementary Table S5: LncRNAs associated with disease free survival

See Supplementary File 5

Supplementary Table S6: Alterations of lncRNAs associated with ER, PR and HER-2

See Supplementary File 6
